# HCl-selective ionization reactions for improved Cl detection robustness in post-inductively coupled plasma chemical ionization

**DOI:** 10.1039/d5ja00483g

**Published:** 2026-03-05

**Authors:** Zahra Afsharsaveh, Kaveh Jorabchi

**Affiliations:** a Department of Chemistry, Georgetown University Washington DC USA kj256@georgetown.edu

## Abstract

Halogen detection, especially in a multielement fashion and with liquid chromatography, is challenging because of the fundamental limitations of ionization in inductively coupled plasma (ICP) mass spectrometry. Post-plasma ionization alleviates these limitations. For Cl detection, plasma-assisted HCl formation followed by chemical ionization using barium-containing reagent ions has been reported, yielding BaCl^+^ and offering simultaneous detection with other halogens (*e.g.* F detection using BaF^+^). However, the broad reactivity of barium-based ionization also makes it susceptible to interference from other plasma-generated species, increasing the potential for ionization suppression, sensitivity loss, and elevated matrix effects. Here, we report the development of HCl-selective post-plasma ionization reactions to improve ionization robustness and matrix tolerance in Cl detection. We evaluate a series of metal ions (M) with aqueous-phase affinity for chloride to determine their potential to form MCl(NO_3_)_*n*_^+^ (*n* = 0–1) in the post-ICP region. We show that metal ions follow the order Pb^2+^, Bi^3+^, Cd^2+^ > In^3+^ > Zn^2+^, Fe^3+^ >> Ba^2+^ in terms of propensity of their reagent ions to react with HCl in the presence of interfering plasma products such as HNO_3_ and HNO_2_. Among the metal ions, Pb^2+^ also offers the highest sensitivity for Cl detection because of efficient reagent ion generation by electrospray ionization. The robustness of PbCl^+^ detection is also tested in P-containing matrices, revealing a 5-fold improvement relative to that with BaCl^+^ and further confirming improved matrix tolerance due to more selective ionization reactions. These studies offer fundamental insights and pave the way towards robust multielement methods for halogen detection in speciation analyses.

## Abstract 2

Tuning the metal ion in nanoESI improves Cl detection sensitivity and matrix tolerance using oxidative plasma and atmospheric-pressure post-ICP ionization.

## Graphical abstract

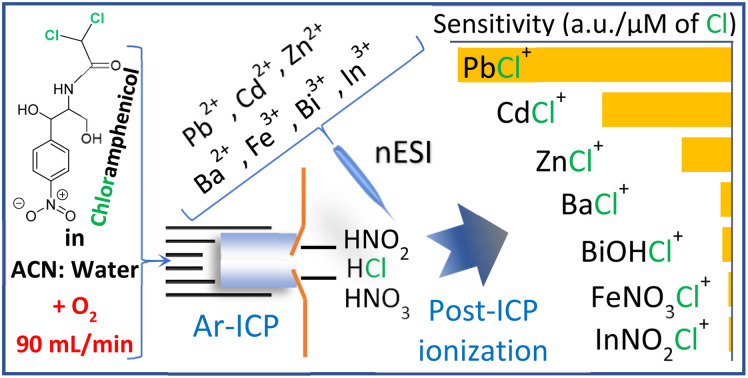

## Introduction

Halogenated compounds have found widespread applications. For example, 81% of commercially launched agrochemicals in 2010–2020 had halogens in their structures with the following breakdown: Br/Cl (2%), Br/F (4%), Cl (10%), Cl/F (25%), F (40%).^1^ Among the 352 drugs approved by the FDA in 2018–2024, 31% were halogenated, with fluorine and chlorine appearing more frequently than other halogens.^2^ On the other hand, halogenated compounds are also regarded as environmental pollutants with chlorinated compounds (*e.g.* disinfection byproducts)^3^ and perfluoroalkyl substances representing two prominent classes of contaminants.

Quantitative and non-targeted halogen detection coupled with liquid chromatography (LC) as well as total halogen detection provides critical insights into the prevalence of halogenated compounds and their transformations in environmental and biological settings.^4^ Accordingly, there has been continuing interest in developing inductively coupled plasma (ICP) mass spectrometry (MS) methods for this purpose, in particular for the detection of chlorinated^5–12^ and fluorinated compounds.^13,14^ There are, however, two main challenges in halogen detection by ICP-MS, especially for F and Cl: (1) inefficient ion formation within the ICP, and (2) isobaric interferences, prevalent at low *m*/*z*. Tandem MS methods (ICP-MS/MS) as well as sector-field ICP-MS^15^ mitigate some of the issues related to the latter challenge while the former challenge continues to persist. The difficulties in ion formation stem from the high ionization potentials of F and Cl along with significant ionization equilibrium shifts by plasma carbon loading when using organic solvents.^6,13^ Moreover, multielement methods that include F detection are challenging with ICP-MS because the current methods use a cool plasma to form the diatomic BaF^+^ within the ICP, while hot plasma conditions are needed for other halogens to maximize atomic ion formation such as Cl^+^. Negative-mode ICP-MS can potentially detect halogens in a multielement fashion; however, ion formation occurs *via* electron capture in the vacuum interface,^16,17^ and remains to be characterized for analytical suitability.

An alternative approach to halogen detection is to implement post-ICP chemical ionization. In this approach, the ICP is utilized as a chemical vapor generator and plasma-produced neutrals are ionized *via* ion-neutral reactions in a cooled post-ICP region. The reagent ions for these ion-neutral reactions can be supplied by a variety of methods such as a nano-electrospray ionization (nESI)^18^ or the plasma itself.^19,20^ One notable ion formation pathway is the plasma-assisted formation of hydrogen halides (HF and HCl) from fluorinated and chlorinated compounds, followed by ionization of these species using reagent ions from nESI of a barium electrolyte, yielding BaF^+^ and BaCl^+^.^18,21^ Unlike in-plasma ionization, this post-ICP chemical ionization approach allows multielement detection of all halogens with common organic solvents for LC (*e.g.* acetonitrile),^21,22^ thanks to decoupling of the plasma from ionization reactions. Further, the approach facilitates coupling of the ion source to ultra-high-resolution Orbitrap instruments, readily resolving isobaric interferences.^21,22^ Therefore, both challenges noted above are mitigated.

New challenges, however, arise in the post-ICP approach. In particular, the broad reactivity of barium-based reagent ions with plasma products, which enables facile multielement detection, also leads to susceptibility of ionization reactions to interferents, and consequently to elevated matrix effects.^22,23^ The ionization interferents may be plasma products from gases and solvents, *e.g.* HNO_3_ produced at high plasma oxygen loads suppressing BaF^+^ and BaCl^+^, or sample-derived species, *e.g.* H_3_PO_4_ from P-containing matrices suppressing BaF^+^ and BaCl^+^, and HCl from Cl-containing matrices suppressing BaF^+^.^18,22^

To alleviate ionization interferences, selective ion-neutral reactions are needed for each element-specific plasma product. To this end, we have reported HF-selective ionization by systematic investigation of metal centers in nESI electrolytes, identifying Sc-based electrolytes for formation of ScFNO_3_^+^ with significantly improved resilience to interference from HNO_3_ and HCl.^23^ Here, we report investigations to improve matrix tolerance and ionization robustness in Cl detection by HCl-selective ionization reactions. These studies complement those of selective HF ionization, and together pave the way toward high-sensitivity and robust multielement methods for speciation of halogenated compounds *via* post-ICP chemical ionization.

## Experimental

### Reagents and sample preparation

Cadmium(ii) nitrate hydrate, lead(ii) nitrate, zinc(ii) nitrate hexahydrate, barium(ii) acetate, iron(iii) nitrate, bismuth(iii) nitrate pentahydrate, and indium(iii) nitrate were purchased from Sigma-Aldrich (Milwaukee, WI). Chloramphenicol, glyphosate, and thiourea with purities ≥98% were acquired from Sigma-Aldrich and were used without further purification. Optima LC-MS grade reagents and solvents, including acetonitrile, formic acid and nitric acid, from Fisher Scientific were used in all experiments. Ultrapure water with a resistivity of 18 MΩ cm was produced from a water purification system (Purelab Flex, ElGA LabWater, Woodridge, IL). All analytical solutions were prepared in a 1 : 1 acetonitrile: water solvent spiked with 0.1% formic acid. The same solvent was used as the flow injection carrier solvent in all experiments.

nESI electrolytes were prepared at 1 mmol L^−1^ concentration in 18 MΩ cm water. For the In(iii) electrolyte, 10 mmol L^−1^ nitric acid was added to the solution while 100 mmol L^−1^ nitric acid was used for Fe(iii) and Bi(iii) electrolytes to facilitate dissolution and to prevent precipitation of hydroxides.

### Sample introduction and ICP parameters

The analytical solutions were injected through a 20-µL PEEK loop at a solvent flow rate of 50 µL min^−1^ supplied by a binary HPLC pump (Vanquish F, Thermo Fisher Scientific, Germany). The solution stream was nebulized into a cyclonic spray chamber using a high-efficiency nebulizer (HEN-90-0.05, Meinhard, Golden, CO) at an argon nebulizer gas flow rate of 1.1 L min^−1^. The spray chamber was cooled to −2 °C by a Peltier cooler (PC3X, Elemental Scientific Inc., Omaha, NE) to reduce solvent vaporization from spray chamber walls. The resulting aerosol was mixed with 0.3 L min^−1^ of makeup argon gas and 90 mL min^−1^ of oxygen gas, and was directed into the ICP *via* a 2.0-mm injector. The argon ICP was sustained using a free-running generator (Nexion, PerkinElmer) at 1300 W with plasma and auxiliary gas flow rates of 14 L min^−1^ and 1.2 L min^−1^, respectively. Note that plasma power has minimal impact on the signal in post-plasma ionization and is not a critical parameter.^24^

### Post-ICP chemical ionization

Plasma-generated species were sampled into a two-chamber chemical ionization interface described in detail previously.^22^ A cartoon representation of the concept is also provided in the graphical abstract. Briefly, the central axis of the torch was aligned with a 4 mm nickel sampling orifice (threaded to a water-cooled plate) located ∼10 mm downstream of the ICP load coil. The sampling orifice also comprised the entrance of the first chamber of the chemical ionization interface. The sampled plasma traversed through a 4-mm i.d., 5-cm long quartz tube, which served as a cooling and recombination area. A set of parallel plate electrodes positioned at the outlet of the quartz tube were biased to a potential difference of 400 V relative to each other, deflecting plasma ions and preventing the ions and electrons from entering the ionization area.

To ionize plasma-produced neutral species, nano-electrospray ionization (nESI) was utilized. The nESI emitter was prepared by pulling a borosilicate glass capillary (1 mm o.d., 0.75 mm i.d., World Precision Instruments, Sarasota, FL) to a tip diameter of 3–4 µm using a micropipette puller (p-87, Sutter Instruments, CA). The emitter was placed in the first chamber downstream of the parallel plate electrodes with the emitter tip recessed approximately 5 mm from the central axis of the quartz tube. A potential of about +1000 V was applied to the electrolyte inside the emitter using a platinum electrode, producing metal-containing reagent ions to react with plasma-generated neutral species. The mixture of the plasma flow and the reagent ions was then transferred to the second chamber through a grounded 1-cm long and 2-mm i.d. stainless steel tube, enhancing mixing and ion-neutral reactions. The second chamber was sealed to the ion sampling plate of the mass spectrometer, allowing the instrument to sample a fraction of the flow emerging from the stainless-steel tube.

The flow of neutrals in the chemical ionization interface was controlled by the input gas flow rate into the first chamber and the gas evacuation rate from the second chamber. These values were optimized at 3.75 L min^−1^ nitrogen and 0.5 L min^−1^ oxygen introduced into the first chamber, while an evacuation rate of 4.2 L min^−1^ was applied to the second chamber, moving the flow toward the MS. Note that the gas sampling by the MS increases the total evacuation rate from the second chamber beyond 4.2 L min^−1^. For optimization, the oxygen flow rate was kept constant while the nitrogen flow rate was scanned to obtain the maximum analytical ion signal. Detection of ZnCl^+^ using an nESI solution containing 1 mmol L^−1^ zinc nitrate along with injection of 20 µmol per L Cl from chloramphenicol was utilized in the optimization experiments. All plasma and interface operating conditions were kept constant for comparison of ionization reactions induced by nESI of various electrolytes.

### MS parameters

The ions were detected using a QExactive Orbitrap instrument (ThermoFisher) operated with a heated capillary temperature of 150 °C and S-lens setting of 50. All detection schemes used an automatic gate control setting of 1 × 10^5^ to prevent overfilling of the Orbitrap. For characterization of ionization reactions, a resolving power setting of 140 000 and maximum injection time of 450 ms were used (except for indium-based analytical ions where resolving power and maximum ion injection time were 35 000 and 160 ms, respectively), and no in-source collision induced dissociation (CID = 0) was applied to preserve and identify the original ions in the ionization area. For characterizing the analytical figures of merit and matrix effects using PbCl^+^, the CID, resolving power, and maximum ion injection time were optimized for best sensitivity, precision, and fastest acquisition.

### Data processing

The.raw files generated by the Orbitrap software were converted to mzml format using Proteowizard software.^25^ The ion intensities were then extracted from mzml files in R^26^ using the MSnbase^27^ package and were processed and graphed by dplyr^28^ and ggplot2 (ref. 29) packages.

## Results and discussion

### Considerations for metal centers in reagent ions for selective and robust HCl ionization

Ion suppression by competing ionization reactions constitutes a major mechanism for matrix effects in post-plasma chemical ionization. In the case of barium-based ionization, our investigations have revealed that BaNO_2_^+^ and BaHCO_2_^+^ serve as the main reagent ions for ionizing HCl to BaCl^+^.^22^ Competing acidic plasma products, especially when produced at high quantities, can lead to depletion of these reagent ions, resulting in suppression of the BaCl^+^ signal. For example, HNO_3_ produced at high plasma oxygen loads and H_3_PO_4_ formed upon injection of large quantities of P-containing compounds react with BaNO_2_^+^ and BaHCO_2_^+^, producing BaNO_3_^+^ and BaH_2_PO_4_^+^ which have lower reactivity with HCl. Accordingly, Cl detection sensitivity is significantly reduced under these conditions.^22^

The matrix effects can also be described by direct reactions of interfering plasma products with the analytical ion, BaCl^+^:

1 BaCl^+^_(g)_ + HA_(g)_ ⇌ BaA^+^_(g)_ + HCl_(g)_

where HA denotes an interfering acidic product with an A^−^ anion. Species with high gas-phase acidity and abundance (*e.g.* HNO_3_) could drive Reaction 1 to the right, thus causing matrix effects and loss of Cl detection sensitivity. To prevent Reaction 1 from proceeding and to minimize matrix effects one can utilize metal centers with higher affinity for Cl^−^, thus creating a thermodynamic barrier for Reaction 1.

Notably, the strategy of metal-center tuning to enhance ionization robustness has been successful in our recent work mitigating matrix effects for F detection *via* HF ionization.^23^ In particular, using Sc(iii) instead of Ba(ii) salts in nESI yields Sc(NO_3_)_2_(H_2_O)_*n*_^+^ ions which lead to analytical ion generation by Reaction 2:^23^

2 Sc(NO_3_)_2_(H_2_O)_*n*_^+^_(g)_ + HF_(g)_ ⇌ ScF(NO_3_)(H_2_O)_*n*_^+^_(g)_ + HNO_3(g)_

where the strength of the Sc–F bond in ScFNO_3_^+^ improves the ionization efficiency in the presence of interfering nitric acid, imparting better tolerance to interferences compared to that offered by BaF^+^ detection. Interestingly, these studies also revealed that HF reaction efficiencies with reagent ions (*e.g.* Reaction 2) using various metal centers correlated with fluoride affinities of the metal ions in aqueous solutions.^23^ The similarity of trends in the gas phase and solution phase may be a result of ion hydration, creating a pseudo-aqueous environment in the gas phase. Regardless of the exact cause, the correlation between gas- and solution-phase behaviors offers an approach to select metal ions to improve matrix tolerance.

**Fig. 1:**
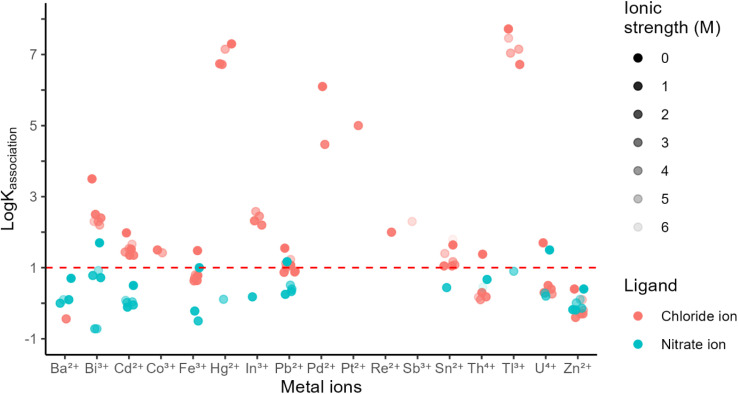
First formation constants of chloro and nitrato complexes for metal ions with log *K*_Cl_ > 1 as well as for Ba^2+^ and Zn^2+^. Formation constant values are taken from ref. 30 and 31.

Similar to Reaction 2, post-plasma ionization of HCl at a high plasma oxygen level (high HNO_3_ production) is expected to proceed *via* Reaction 3:

3 M(NO_3_)_*x*_(H_2_O)_*n*_^+^ + HCl_(g)_ ⇌ MCl(NO_3_)_*x*−1_(H_2_O)_*n*_^+^_(g)_ + HNO_3(g)_

where *x* = 1 and 2 correspond to divalent and trivalent metal centers, respectively. Monovalent metal ions are not considered as they would result in a neutral product (MCl) undetectable with MS. Noting the correlation between solution- and gas-phase behaviors of the ions discussed above, we examined the first formation constants of metal-chloro complexes in solution within a NIST database,^30^ recently repackaged for Windows 7 and later,^31^ to identify the most promising metal centers. Fig. 1 includes metal ions with log *K*_Cl_ > 1 at any reported ionic strength in the database, denoting metals with high affinity for chloride to improve ion formation *via* Reaction 3. Formation constants for other metal ions are shown in Fig. S1. We then applied a set of selection criteria based on a few practical considerations.

Hg^2+^ and Tl^3+^ have high toxicity and were not considered in our studies. nESI from Pd(ii) nitrate solutions were unsuccessful because of frequent tip clogging consistent with the high affinity of Pd^2+^ for hydroxide (log *β*_2_ = 28),^30,31^ which promotes the formation of scarcely soluble Pd(OH)_2_. Similarly, we did not consider Pt^2+^ because of its high affinity for hydroxide (log *β*_2_ = 30).^32^ Among the remaining metal ions with high affinity to chloride (log *K*_Cl_ above the dashed line) we selected Bi^3+^, In^3+^, Fe^3+^, Cd^2+^, and Pb^2+^ based on a clearer preference for chloride over nitrate as well as ease of access to non-chloride, stable, and soluble salts (preferably nitrate salts). In addition to these metal ions, we also included Ba^2+^ with low affinity for chloride relative to nitrate and Zn^2+^ with similar affinities for the two ligands to further expand the range of explored metal characteristics and to facilitate the comparison between solution and gas phase behaviors as discussed below. Inclusion of Ba^2+^ is also helpful for comparison to previous studies of post-ICP ionization with BaCl^+^ detection.^22^

### Interaction of nESI ions with a plasma afterglow and relation between gas-phase and solution-phase behavior

**Fig. 2:**
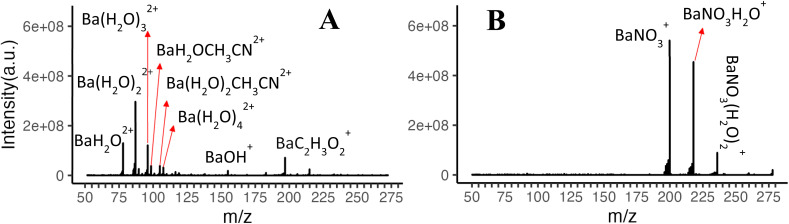
Ions produced by nESI of 1 mmol L^−1^ barium acetate (A) in the absence of plasma (average of 110 scans) and (B) upon sampling plasma into the ionization interface (average of 300 scans).

Ions produced by nESI are modified by subsequent interactions with plasma products (*e.g.* HNO_3_) in the afterglow, giving rise to the reagent ions for HCl ionization (Reaction 3). To gain insights into the type of ions produced by nESI for each metal center and to identify plasma products that modify these ions, we examined the ions before and after interaction with the plasma afterglow. Fig. 2A and B show the results for Ba(ii) electrolyte as a reference point, indicating formation of Ba(H_2_O)_*n*_^2+^ as the main ion by nESI, which is converted to BaNO_3_(H_2_O)_*n*_^+^ upon interaction with the afterglow. This conversion indicates abundant HNO_3_ in the afterglow of an oxygen-loaded Ar-ICP, consistent with our previous findings.^22,23^

**Fig. 3:**
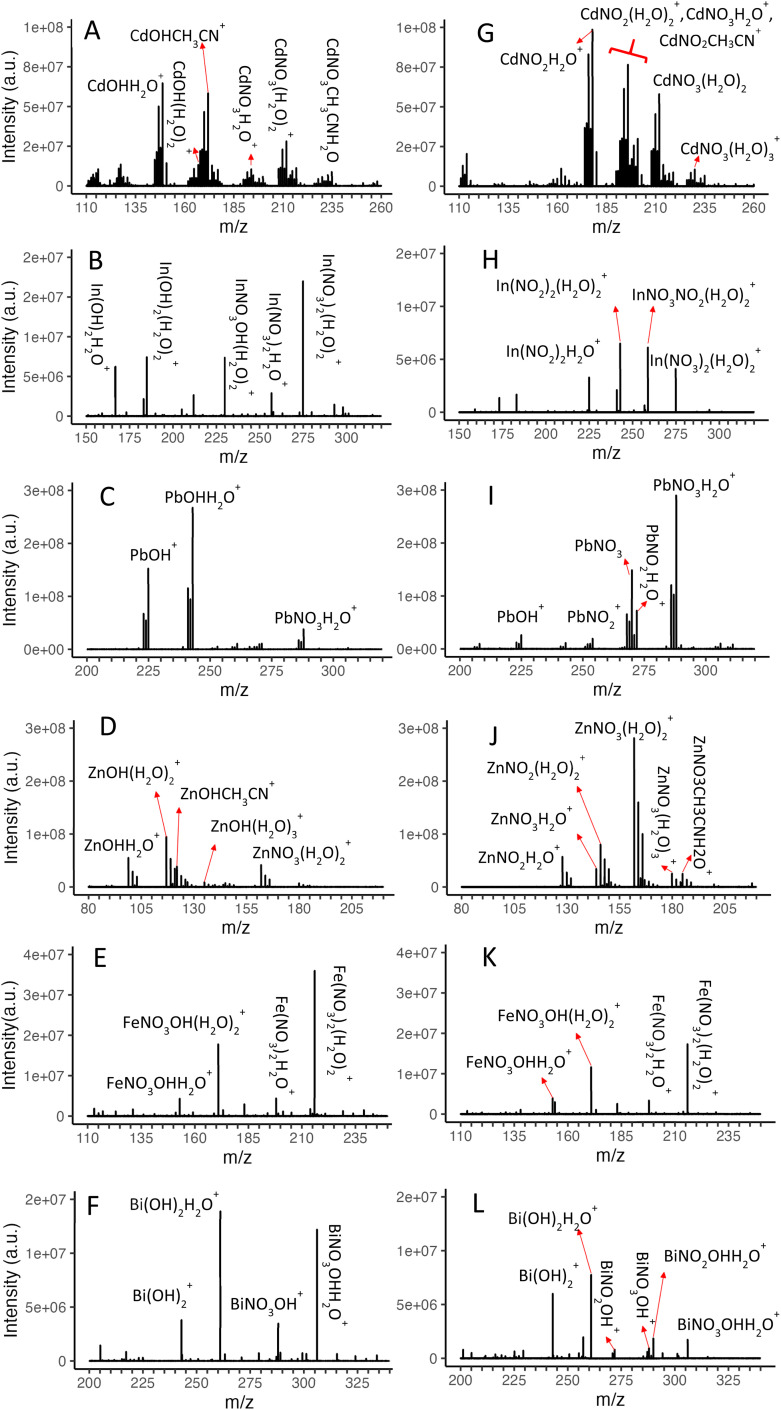
Ions generated from nESI of 1 mmol L^−1^ In^3+^, Zn^2+^, Pb^2+^, Cd^2+^, Bi^3+^, and Fe^3+^ electrolytes in the absence of plasma (A to F), and upon interaction with the plasma afterglow (G to L). Note the variation in the *y* axis scale among various metal ions, reflecting the ion generation efficiency of nESI. Acetonitrile adducts are a result of residual solvent in the chemical ionization interface. All spectra shown here were obtained by averaging 100–200 spectra using a single nESI emitter for each metal ion.

Similarly, Fig. 3A–F display the ions produced by 1 mmol L^−1^ aqueous solutions of Cd^2+^, Zn^2+^, Pb^2+^, Bi^3+^, Fe^3+^ and In^3+^ nitrate salts before interaction with the plasma afterglow. We note that 100 mmol L^−1^ HNO_3_ was added to the solutions of Bi^3+^ and Fe^3+^, and 10 mmol L^−1^ HNO_3_ was added to the In^3+^ solution to prevent extensive hydrolysis of these ions in solution for stable nESI operation. Unlike the case of Ba^2+^, only singly charged ions are observed from the nESI of these metal salt solutions. The high abundance of hydroxo ions indicates the low affinities of the metal ions to nitrate in solution. This is particularly notable for In^3+^, Bi^3+^ and Fe^3+^, where a significant amount of nitric acid is also added to the solutions, increasing the nitrate ion concentration. While hydroxo ions may form in solution (particularly for trivalent ions with high hydroxide affinity, see Table 1), the abundance of hydroxo ions in the spectra may not necessarily reflect the abundance of species in solution. For example, considering a 1 mmol L^−1^ Pb(NO_3_)_2_ solution and formation constants listed in Table 1, one would expect more than 95% of Pb^2+^ to exist as doubly charged ions while PbNO_3_^+^ would have about 3 fold higher concentration than that of PbOH^+^. The high prevalence of hydroxo ions in the spectrum of Pb^2+^ electrolyte therefore indicates promoted hydrolysis during the charge reduction process of droplets in electrospray ionization.^33^

**Table 1:** The gas-phase reactivity of reagent ions with HCl and solution-phase formation constants of metal ions with OH^−^, NO_3_^−^, NO_2_^−^, and Cl^−^a

Metal ion	Reagent ion reactivity with HCl in the gas phase (1/(µmol L^−1^))	Analytical ion	Reagent ions	log *K*_association_ in the solution phase^30,31^			
				Cl^−^	OH^−^	NO_3_^−^	NO_2_^−^
Pb^2+^	1.38 × 10^−4^ ± 2.8 × 10^−6^	PbCl(H_2_O)_*n* = 0–2_^+^	PbNO_2_(H_2_O)_*n* = 0–1_^+^	1.55 (0)	6.40 (0)	1.17 (0)	2.51 (0)
			PbNO_3_(H_2_O)_*n* = 0–1_^+^				
			PbOH^+^				
Bi^3+^	1.32 × 10^−4^ ± 1.23 × 10^−5^	BiClOH(H_2_O)_*n* = 0–2_^+^	Bi(OH)_2_(H_2_O)_*n* = 0–1_^+^	2.5 (0.5)	12.30 (0.5)	0.72 (0.5)	NA
			BiOHNO_2_(H_2_O)_*n* = 0–1_^+^				
			BiOHNO_3_(H_2_O)_*n* = 0–1_^+^				
Cd^2+^	1.07 × 10^−4^ ± 1.9 ×10^−6^	CdCl(H_2_O)_*n* = 0–2_^+^	CdNO_2_(H_2_O)_*n* = 1–2_^+^	1.54 (3)	4.30 (3)	0.04 (3)	1.80 (3)
			CdNO_3_(H_2_O)_*n* = 1–3_^+^				
			CdNO_2_CH_3_CN^+^				
In^3+^	4.54 × 10^−5^ ± 5.3 × 10^−6^	InClNO_2_(H_2_O)_*n* = 1–2_^+^	In(NO_2_)_2_(H_2_O)_*n* = 1–2_^+^	2.32 (0.7)	10.07 (0)	0.18 (0.7)	2.6 (1)
			InNO_2_NO_3_(H_2_O)_2_				
Zn^2+^	2.82 × 10^−5^ ± 1.6 ×10^−6^	ZnCl(H_2_O)_*n* = 1–2_^+^	ZnNO_2_(H_2_O)_*n* = 1–2_^+^	−0.3 (1)	4.7 (1)	−0.19 (1)	0.37 (1)
			ZnNO_3_(H_2_O)_*n* = 1–3_^+^				
			ZnNO_3_H_2_OCH_3_CN^+^				
Fe^3+^	2.27 × 10^−5^ ± 8.7 × 10^−7^	FeClNO_3_(H_2_O)_2_^+^	FeOHNO_3_(H_2_O)_*n* = 1–2_^+^	0.63 (1)	11.05 (1)	−0.5 (1)	2.59 (1)
			Fe(NO_3_)_2_(H_2_O)_*n* = 1–2_^+^				
Ba^2+^	3.44 × 10^−6^ ± 3.5 × 10^−7^	BaCl(H_2_O)_*n* = 0–1_^+^	BaNO_3_(H_2_O)_*n* = 0–2_^+^	−0.44 (1)	0.64 (0)	0 (1)	NA

a The error for gas-phase reactivity reflects the standard deviation of three measurements where each measurement was obtained by using a freshly made nESI emitter and averaging triplicate injections of 20 µmol per L Cl from chloramphenicol. The numbers in parentheses reflect ionic strength (mol L^−1^) at 25 °C for indicated log *K* values.

The interactions of nESI ions with plasma flow also provide insights about plasma-produced species as shown in Fig. 3G–L. In contrast to observations with Ba(ii) electrolyte where BaNO_3_^+^ is the only species detected upon the interaction of nESI ions with the plasma flow, nitrito complexes are detected in the majority of cases for other metals, indicating the presence of HNO_2_ in the post-plasma flow in addition to HNO_3_. The relative concentrations of HNO_3_ and HNO_2_ are not known in our experiments but they remain constant as the experiments are conducted under constant plasma operating and plasma sampling conditions. Therefore, the relative abundances of nitrito and nitrato ions in the spectra can be used to infer selectivity of the original nESI ions for reactions with HNO_2_*versus* HNO_3_.

The metal ions can be broadly placed in three categories based on conversion of the original hydroxo ions to new products upon interaction with post-plasma flow: (1) for Cd(ii) and In(iii), the original hydroxo ions are depleted and nitrito ions dominate the nESI-plasma interaction products. This suggests a higher selectivity of the nESI ions of these metals for reaction with HNO_2_. (2) For Pb(ii) and Zn(ii), the hydroxo ions are depleted and nitrato ions dominate the product spectrum. These metals therefore show a reduced affinity for HNO_2_ compared to those of Cd(ii) and In(iii). (3) For Bi(iii) and Fe(iii), the original hydroxo ions remain abundant after interaction with the plasma flow, indicating low affinity of these ions to reaction with both HNO_3_ and HNO_2_ in the gas phase.

The categories above draw parallels to reactions of metal ions in the solution phase. Table 1 lists the first formation constants of metal ions with hydroxide, nitrite, and nitrate in solution (selected at similar ionic strengths for each anion) for comparison to the behavior observed in the gas phase. Bi(iii) and Fe(iii) have the highest affinity for hydroxide compared to other anions, in agreement with the low reactivity of nESI ions from these metals with HNO_2_ and HNO_3_ in the gas phase (category 3 above). Cd(ii) and In(iii) have higher affinities for nitrite compared to nitrate in solution, consistent with increased selectivity of the hydroxo ions of these metals to reaction with HNO_2_ as observed in the spectra (category 1 above). Finally, the preference of Pb(ii) and Zn(ii) for reaction with nitrite *versus* nitrate in solutions is lower than those of Cd(ii) and In(iii), which is also consistent with the higher nitrato products observed for Pb(ii) and Zn(ii). Overall, a general similarity between behaviors in the gas phase and solution phase is observed for reactions with acidic plasma products, further reinforcing the strategy of selecting metal centers based on chloride solution phase affinities for selective gas-phase ionization of HCl.

### Post-plasma ionization of HCl in the presence of interferents

The reactions of reagent ions with HCl in the presence of HNO_3_ and HNO_2_ were investigated *via* flow injections of 20 µmol per L Cl from chloramphenicol. Table 1 lists the analytical and corresponding reagent ions identified as discussed below.

For divalent metals, MCl(H_2_O)_*n*_^+^ is the only possible analytical ion. The *n* values were selected to include hydration levels with ion intensities exceeding 2% of the most abundant hydration level. In the case of barium, this threshold was 10% due to low intensities of the ions. This selection process ensured proper inclusion of the hydration distribution for each ion. Monoisotopic *m*/*z* intensities of the selected hydration levels were measured within 1-*m*/*z* wide SIM windows. Flow injection peak heights across the monitored hydration levels were then summed to calculate the total analytical ion intensity.

For trivalent metals, a variety of analytical ions represented by MClX(H_2_O)_*n*_^+^ are plausible where X denotes OH, NO_2_ and NO_3_. To select the most promising species, we first conducted scouting experiments by monitoring the monoisotopic *m*/*z* of each species with various hydration levels using 1-*m*/*z* wide SIM windows and flow injection of chloramphenicol. Only ions with detectable intensities (listed in Table 1) were then selected to build multi-SIM window methods for further investigations. Trivalent metals generally produce lower intensities of ions. Therefore, the *n* values for hydration levels were selected to capture species with intensities reaching 10% of the most abundant hydration level. Similar to divalent metals, the flow injection peak heights across hydration levels were summed to obtain the total analytical ion intensity. Note that monoisotopic *m*/*z* also corresponds to the most abundant isotopologue of all species in this work.

To compare the performances of various metal ions, we considered two factors: (1) sensitivity: defined as the total intensity of analytical ions (summed over hydration levels) per µmol L^−1^ of Cl injected. The higher sensitivities generally offer better analytical performance. (2) Reactivity of reagent ions: this property reflects the efficiency of reactions between HCl and reagent ions and can be quantified by eqn (4):

4 Reactivity = sensitivity/total reagent ion intensity

Considering that the consumption of reagent ions upon reaction with HCl is negligible, evident from orders of magnitude lower analytical ion intensity compared to that of reagent ions, the ratio in eqn (4) yields the fraction of reagent ions converted to analytical ions upon reaction with HCl. Higher reactivities in the presence of HNO_2_ and HNO_3_ interferents indicate better thermodynamic favorability of ionization (*e.g.* by Reaction 3), improved selectivity, and reduced matrix effects.

Since the exact reagent ion for reacting with HCl was not identified, the sum of the intensities for reagent ions indicated in Table 1 was used in reactivity calculations for each analytical ion. Further, reagent ions with intensities reaching 10% of the most abundant species in the spectra of Fig. 3 were considered in these analyses. Similar to analytical ions, the monoisotopic *m*/*z* of each reagent ion was used in the calculations, thus mitigating the effect of isotopic distribution on the calculated reactivity.

We first consider reactivity because of its relevance to improved matrix tolerance. Table 1 lists the metal ions in the order of reactivities: Pb^2+^, Bi^3+^, Cd^2+^ > In^3+^ > Zn^2+^, Fe^3+^ >> Ba^2+^. The aggregate rather than reagent ion-specific nature of the calculated reactivity and presence of both HNO_2_ and HNO_3_ as interferents with unknown concentrations in the ionization area make the reactivity trend between the metals difficult to interpret. Nevertheless, the new metal ions show substantial (over 10-fold) improvement in reactivity compared to that of Ba(ii), in line with expectations from solution phase affinity for chloride *versus* nitrate. Overall, the reactivity results above indicate the potential of Bi(iii), Pb(ii), and Cd(ii) as preferred metal centers to increase the selectivity of ionization and minimize matrix effects in Cl detection by post-plasma chemical ionization.

**Fig. 4:**
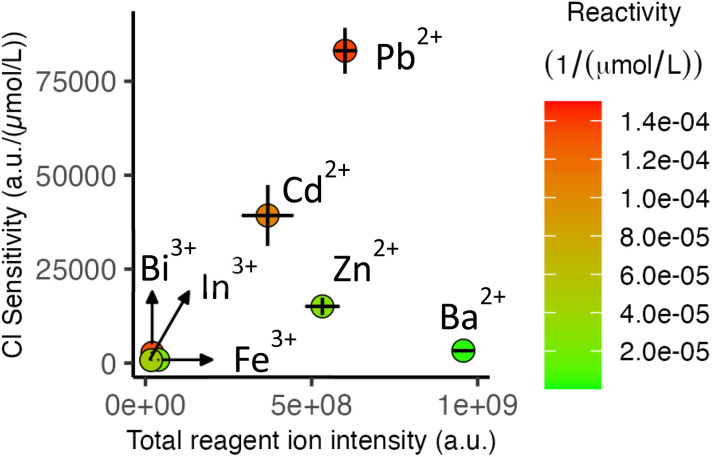
Relation of Cl detection sensitivity with abundance and reactivity of reagent ions. Note that the monoisotopic *m*/*z* (also corresponding to the most abundant isotopologue) for reagent ions and analytical ions noted in Table 1 are used in this analysis. The error bars represent standard deviations based on measurements from three nESI emitters and triplicate injections using each emitter.

As discussed above, sensitivity constitutes another important factor for analytical performance. Fig. 4 depicts the relations between sensitivity, total reagent ion intensity, and reactivity. Metal centers with low-reactivity reagent ions (Fe^3+^, Zn^2+^, and Ba^2+^ denoted by green color and located along the *x* axis of Fig. 4) result in low sensitivity regardless of reagent ion abundance. In fact, Ba^2+^ provides the highest reagent ion intensity among all metal ions, and yet leads to low sensitivity because of inefficient reactions in the presence of interferents. In contrast, for metal centers with reagent ions of high reactivity (Bi(iii), Cd(ii), Pb(ii)), Cl detection sensitivity is determined by the ability to create abundant reagent ions. This is evident from the near-linear relationship in Fig. 4 for Bi(iii), Cd(ii), and Pb(ii).

Fig. 4 also shows that trivalent ions produce a lower intensity of reagent ions compared to divalent ions (also evident in the spectra in Fig. 3), indicating a lower ion generation efficiency for nESI. This is likely because of the high propensities of trivalent ions for hydrolysis, which also required the addition of HNO_3_ to the solutions. The added acid produces a competition between metal ions and protons in the ESI process, reducing reagent ion generation efficiency. Moreover, metal centers with more isotopes result in splitting of the nESI current between multiple *m*/*z* values, reducing the intensity of the monoisotopic *m*/*z*. For example, ^208^Pb^35^Cl^+^ accounts for 40% of the isotopic distribution while ^114^Cd^35^Cl^+^ accounts for 22% of the distribution. Interestingly, the near two-fold Cl sensitivity difference between Pb^2+^ and Cd^2+^ in Fig. 4 is close to the factor of 1.8 expected from the isotopic envelopes of PbCl^+^ and CdCl^+^ noted above, suggesting that the difference in sensitivity using the two metals is largely a result of their different isotopic distributions.

Overall, considerations of reactivity and sensitivity highlight Pb(ii) as a favorable metal among the tested metal ions for Cl detection *via* post-ICP HCl ionization.

### Linearity and limit of detection (LOD) using PbCl^+^

To further evaluate the analytical potential of PbCl^+^ detection, we measured linearity, limit of detection (LOD), and background equivalent concentration (BEC). To achieve the best detection capabilities using the Orbitrap instrument, the CID was optimized (60 V) to decluster the hydrated ions. A 1-m/z-wide window around the most abundant isotopologue of PbCl^+^ was selected using the quadrupole filter and the lowest resolving power setting of 17 500 was applied to increase acquisition frequency which would be needed to capture transient signals in applications such as chromatography. Note that ^208^Pb^35^Cl^+^ and ^206^Pb^37^Cl^+^ are not separated at this resolution setting.

The measurements were conducted at a maximum ion trap fill time of 160 ms with an automatic gain control of 1 × 10^5^ to allow sufficient ions to accumulate prior to the injection and to avoid overfilling the trap during the injection. The ion intensities did not reach the AGC threshold in 160 ms prior to the injections, effectively setting the integration time to 160 ms during the baseline measurement time (prior to injection).

**Fig. 5:**
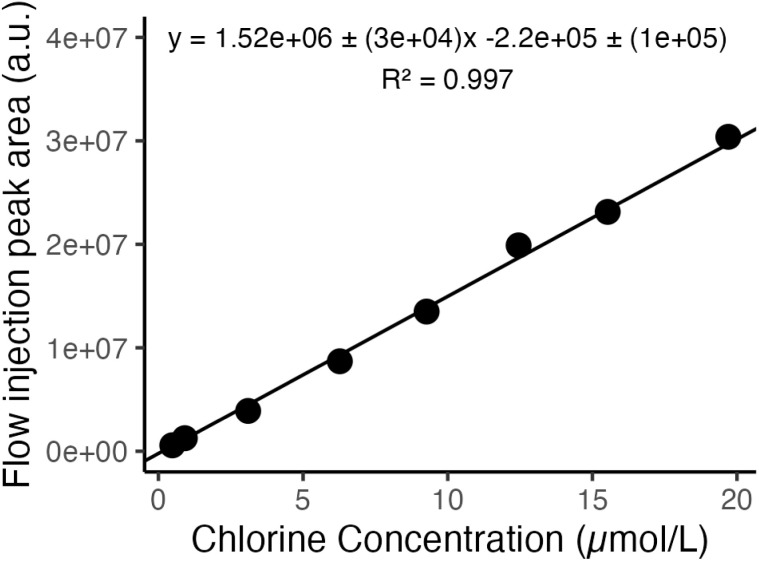
Calibration curve for Cl detection as PbCl^+^ by flow injection of chloramphenicol weighted by 1/peak area.

The analytical figures of merit were measured by constructing a calibration curve using flow injections of chloramphenicol solutions with elemental Cl concentrations of 0.5–20 µmol L^−1^. Fig. 5 shows the curve based on flow injection peak areas, demonstrating good linearity. The limit of detection for chlorine was determined from 3× noise/sensitivity where sensitivity was obtained from the slope of the calibration curve using flow injection peak heights (Fig. S2). The noise was defined as the standard deviation of the baseline prior to injection, while the BEC was determined based on the average baseline intensity divided by sensitivity.

These experiments resulted in an LOD of 0.25 µmol L^−1^ (8.9 ng per mL Cl) and BEC of 2.7 µmol L^−1^ (96 ng per mL Cl). Table 2 compares these results to those of post-plasma ionization using barium electrolyte and ICP-MS/MS. Note that the measurements using Ba(ii) were conducted at low plasma oxygen levels and higher plasma carbon loading (solvent flow rate) to minimize the formation of HNO_3_. Compared to using BaCl^+^, a higher LOD is observed with PbCl^+^. However, the current study is performed at a lower integration time. Increasing the integration time to 1000 ms would decrease the LOD by 2.5-fold. With a correction of 2.5-fold for the current work, the LODs of the two post-ICP ionization approaches are at ∼3.5% of BEC, indicating that the main contributor to the higher LOD with PbCl^+^ is the higher BEC from increased Cl contamination. One contributor to BEC is the trace chloride in the nESI electrolyte, which would have a higher impact using Pb(ii) given the higher affinity of this ion for chloride compared to that of Ba(ii) in the nESI solution. It is also of note that LODs for ICP-nESI-HR-MS with PbCl^+^ and Cl^+^ detection by ICP-MS/MS are approximately at 10% of their corresponding BECs, suggesting comparable precision and dominance of BEC in determining the LOD. Efforts to reduce BEC, *e.g.* use of higher purity and chloride-free metal salts as well as lower nESI electrolyte concentrations, can improve performance and provide better LODs. Moreover, it is notable that post-plasma ionization offers more facile multielement detection of nonmetals (particularly when combined with F detection) compared to other plasma-based techniques as further elaborated on in the conclusions.

**Table 2:** LOD and BEC using PbCl^+^ for Cl detection and comparison to other methods

Method	Ion and MS/MS transition	LOD (ng per mL Cl)	BEC (ng per mL Cl)	Solvent and flow rate	Integration time (ms)	Ref.
ICP-nESI-HR-MS (90 mL per min O_2_)	PbCl^+^	8.9	96	50% CH_3_CN in H_2_O + 0.1% formic acid, 50 µL min^−1^	160	This work
ICP-nESI-HR-MS (45 mL per min O_2_)	BaCl^+^	0.6	17	50% CH_3_CN in H_2_O + 0.1% formic acid, 200 µL min^−1^	1000	22
ICP-MS/MS	Cl^+^ + H_2_ → ClH_2_^+^	1	N.A.	CH_3_OH + 0.1% formic acid, 1000 µL min^−1^	100	34
LC-ICP-MS/MS	Cl^+^ + H_2_ → ClH_2_^+^	∼1^a^	∼10^b^	Water + 0.15% trifluoroacetic acid, 800 µL min^−1^	N.A.	7

a This study uses LC. For comparison to other methods within the Table, the 1.4–1.6 ng per mL Cl LODs reported in the study^7^ are scaled by 0.7 to account for lower concentration at the apex of the LC peak compared to the concentration in the injected sample. The scaling factor is calculated by estimating a full width at half maximum of 0.08 min from the chromatograms in Fig. 1B of the study, assuming a gaussian peak shape, and considering a 50 µL injection at 0.8 mL min^−1^ flow rate.

b BEC is estimated from the peak height to baseline ratio in Fig. 1B of ref. 7 and consideration of the 0.7 scaling factor noted above.

### Matrix effect from S and P for the detection of Cl as PbCl^+^

To further characterize the matrix tolerance using PbCl^+^ detection, we evaluated effects from sulfur and phosphorus. These nonmetal elements are prevalent in biological samples and produce acidic plasma products in oxidative conditions, namely H_2_SO_4_ and H_3_PO_4_, posing potential interference for HCl ionization. PbCl^+^ intensity as well as intensities of reagent ions were monitored upon injection of chloramphenicol with and without S- and P-containing compounds spiked in solutions. The analytical ion (PbCl^+^) was monitored using a 1-*m*/*z* window at CID = 60, resolving power of 35 000, and maximum injection time of 125 ms, while the reagent ions were monitored in a 50–500 *m*/*z* window with CID = 20 (to enhance dehydration), resolving power of 140 000 and maximum injection time of 450 ms. The most abundant isotopologue of each dehydrated reagent ion was used for the analyses.

To quantify the matrix effect in Cl detection, the PbCl^+^ response factor (flow injection peak area per Cl concentration) was normalized to that obtained without a matrix. The reagent ion intensities detected at the apex of the flow injection peak were normalized to those at the base prior to the peak. The results of these experiments are summarized in Table 3. For comparison, effects of S and P matrices on Cl detection *via* BaCl^+^ reported in our previous work^22^ are tabulated in Table 4. Please note that the experiments using BaCl^+^ in the previous work were conducted at low oxygen levels (20 mL min^−1^) as noted previously due to severe interference from HNO_3_ at high oxygen loadings.

**Table 3:** Effect of sulfur and phosphorus matrices on detection of Cl as PbCl^+^^a^

Cl concentration (µmol L^−1^) from chloramphenicol	Matrix concentration	Normalized PbCl^+^ response factor (%)	Ratio of reagent ion intensity at the flow injection peak apex to that at the base		
			PbOH^+^	PbNO_2_^+^	PbNO_3_^+^
21.5	No matrix	100 ± 4	0.99 ± 0.02	0.98 ± 0.06	0.99 ± 0.04
21	9.9 mmol per L S from thiourea	97 ± 4	1.02 ± 0.03	0.94 ± 0.01	0.95 ± 0.06
19.6	97 µmol per L P from glyphosate	97 ± 5	0.90 ± 0.01	0.90 ± 0.03	0.92 ± 0.02
22.4	507 µmol per L P from glyphosate	91 ± 3	0.69 ± 0.03	0.71 ± 0.04	0.70 ± 0.02

a Errors reflect the standard deviation of triplicate injections.

**Table 4:** Effect of sulfur and phosphorus matrices on detection of Cl as BaCl^+^ measured at a low plasma oxygen level of 20 mL min^−1^ (data taken from ref. 22)

Cl concentration (µmol L^−1^) from chloramphenicol	Matrix concentration	Normalized BaCl^+^ response factor (%)
3.1	No matrix	100 ± 3
3.1	127 µmol per L P from glyphosate	71 ± 2
3.1	6.6 mmol per L S from thiourea	90 ± 2

The comparison of data in Tables 3 and 4 shows that a negligible change in PbCl^+^ detection is recorded with the 9.9 mmol per L S matrix while the 6.6 mmol per L S matrix produced a 10% suppression of BaCl^+^ signal. Similarly, the 97 µmol per L P matrix resulted in no discernable signal change for PbCl^+^ while the 127 µmol per L P matrix led to ∼30% suppression of BaCl^+^ intensity. Additionally, the 500 µmol per L P matrix produced only a 10% suppression of the PbCl^+^ signal, indicating an over 5-fold improvement in matrix tolerance compared to BaCl^+^. These results highlight the improved matrix tolerance using lead-based reagent ions for chlorine detection compared to that with BaCl^+^.

Interestingly, the reagent ions in Table 3 are suppressed to a greater extent than PbCl^+^ by the P matrix. Further, PbH_2_PO_4_^+^ is detected at *m*/*z* 304.9406 upon injection of the P-containing matrix (Fig. S3), indicating conversion of reagent ions to PbH_2_PO_4_^+^*via* reactions with H_3_PO_4_ produced by the plasma. The lower extent of suppression for PbCl^+^ compared to reagent ions suggests that PbH_2_PO_4_^+^ may serve as a reagent ion to form PbCl^+^ from HCl, but with lower reactivity compared to the original reagent ions.

## Conclusions

Here, we demonstrate a systematic approach to improve the analytical performance of Cl detection in post-plasma chemical ionization by tuning reagent ions for selective reactions with HCl. This tuning was guided by solution-phase affinities of metal centers for chloride compared to other anions. Metal centers were first selected based on their potential to minimize interference from HNO_3_, which forms in abundance from an oxygen-loaded Ar-ICP. We observed that reagent ion interactions with the major post-plasma species generally followed those expected from solution-phase trends. Notably, these experiments also revealed that HNO_2_ is generated by the plasma even at high oxygen levels and may act as an interferent depending on the metal center (*e.g.* lower reactivity observed with In(iii) despite high affinity for chloride based on the solution phase).

Among the metals that show high affinity for chloride in solution, Pb^2+^ emerged as a preferred choice because of several factors: (1) high reactivity of produced reagent ions with HCl in the presence of HNO_3_ and HNO_2_, (2) low hydrolysis propensity of Pb^2+^ in solution, resulting in good reagent ion formation efficiency in the ESI process, and (3) limited isotopic distribution, concentrating the nanospray ions into fewer ions and increasing sensitivity. A comparison of matrix effects, particularly from P, also confirmed that Pb^2+^ as the metal center in reagent ions significantly improves the robustness of Cl detection compared to that with Ba^2+^, thanks to increased selectivity for reactions with HCl.

The detection limits for Cl in this work are higher than those reported by ICP-MS/MS; however, the dominant factor in this trend is the high BEC from Cl contamination rather than fundamental shortcomings, which can be remedied by practical considerations. Importantly, post-plasma chemical ionization offers multielement detection of nonmetals, a fundamental advantage over other plasma-based methods, thanks to decoupling of ionization from plasma temperature. This capability was demonstrated using Ba-based ionization in our previous work for F, Cl, Br, I, S, and P detection.^22^ However, the ionization was more prone to matrix effects, and plasma oxygen synchronization was needed with gradient chromatography to avoid HNO_3_ interference, complicating operation.^21^ The current work mitigates these drawbacks for Cl detection. Further, the improved reactions for Cl detection can be integrated with F detection by Sc(iii)-based ionization^23^*via* mixed electrolyte nESI, enabling multielement and robust halogen detection readily interfaceable with reversed phase chromatography. The works in these areas are currently under development in our laboratory.

## Author contributions

Zahra Afsharsaveh: methodology, investigation, validation, formal analysis, writing – original draft, visualization. Kaveh Jorabchi: conceptualization, methodology, formal analysis, writing – review and editing, visualization, supervision, project administration, funding acquisition.

## Conflicts of interest

Kaveh Jorabchi is a co-inventor of granted and pending US and European patents related to the technique reported here.

## Supplementary Material

JA-041-D5JA00483G-s001

## Data Availability

All data in the manuscript are accessible from the Figshare repository *via*https://doi.org/10.6084/m9.figshare.30727436. Supplementary information (SI) is available. See DOI: https://doi.org/10.1039/d5ja00483g.
