# Supplementary material for: HCl-selective ionization reactions for improved Cl detection robustness in post-inductively coupled plasma chemical ionization
Source: J Anal At Spectrom. 2026 Mar 5;41(5):1701–12. doi: 10.1039/d5ja00483g (PMC13006787; doi:10.1039/d5ja00483g)
Supplement: JA-041-D5JA00483G-s001 [file JA-041-D5JA00483G-s001.pdf]

Supplementary Information (SI) for Journal of Analytical Atomic Spectrometry.
This journal is © The Royal Society of Chemistry 2026

Supporting information

HCl-selective ionization reactions for improved Cl detection robustness in post-inductively coupled plasma chemical ionization

Zahra Afsharsaveh and Kaveh Jorabchi*

Department of Chemistry, Georgetown University, Washington, DC

* Corresponding author: kj256@georgetown.edu

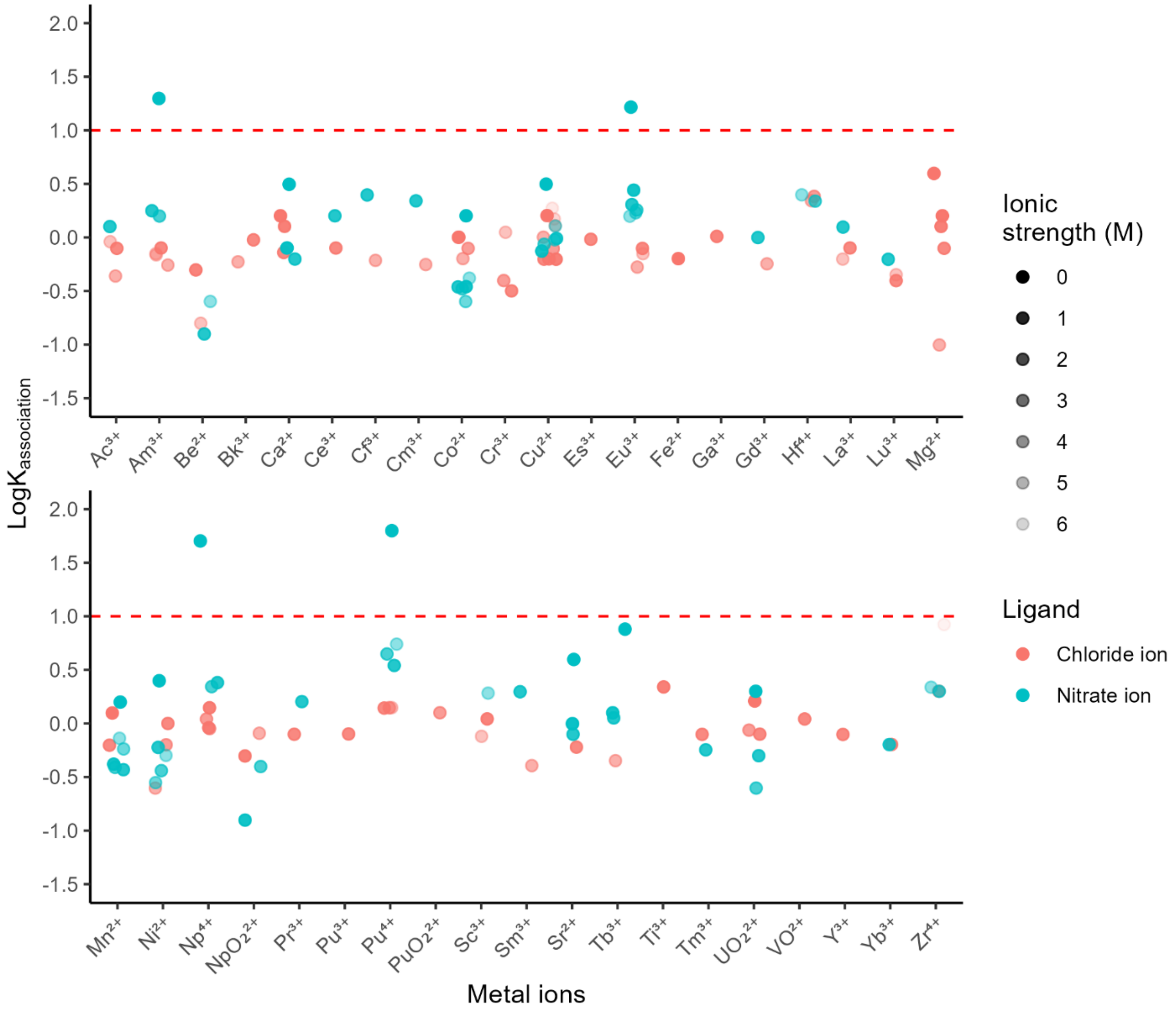

Figure S1. First formation constants of chloro and nitrato complexes for metal ions with LogK$_{Cl}$ < 1. LogK$_{Cl}$ values are taken from References 1,2.

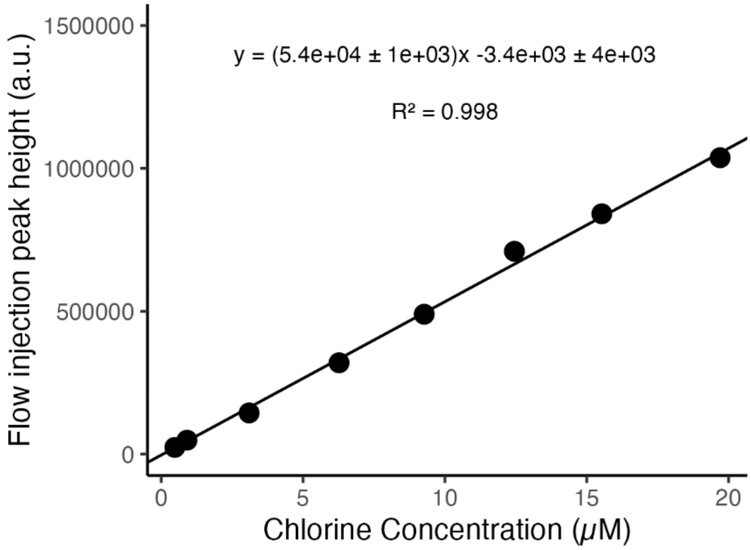

Figure S2. Calibration curve for Cl detection as $PbCl^+$ using flow injection peak heights and chloramphenicol as analyte. Regression line is weighted by 1/peak height.

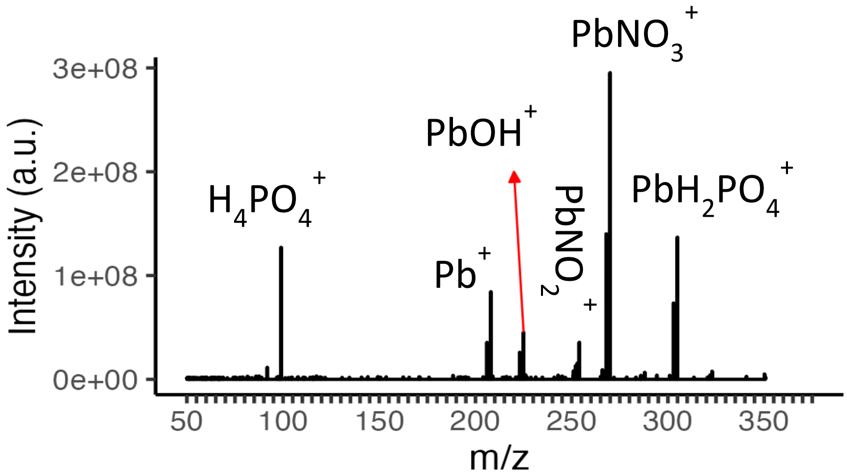

Figure S3. Pb-containing ions detected upon injection of 500 μM glyphosate. CID for this experiment was set to 20 to decluster hydrated ions.

References

1 Burgess, Donald, R., NIST SRD 46. Critically Selected Stability Constants of Metal Complexes: Version 8.0 for Windows, National Institute of Standards and Technology, 2004 DOI: 10.18434/M32154 (accessed 2025-11-13).

2 Hatada, Naoyuki, Stability Constant Explorer - Database of Stability Constants of Metal Complexes (version v1.1.2) https://n-hatada.github.io/stability-constant-explorer/english.html 2023.
